# Non-tuberculous mycobacteria have diverse effects on BCG efficacy against *Mycobacterium tuberculosis*^☆^

**DOI:** 10.1016/j.tube.2013.12.006

**Published:** 2014-05

**Authors:** Hazel C. Poyntz, Elena Stylianou, Kristin L. Griffiths, Leanne Marsay, Anna M. Checkley, Helen McShane

**Affiliations:** The Jenner Institute, Nuffield Department of Clinical Medicine, University of Oxford, Old Road Campus Research Building, Oxford OX3 7DQ, United Kingdom

**Keywords:** Bacillus Calmette-Geurin, Non-tuberculous mycobacteria, *Mycobacterium avium*, Tuberculosis, Mouse model

## Abstract

The efficacy of Bacillus Calmette-Guerin (BCG) vaccination in protection against pulmonary tuberculosis (TB) is highly variable between populations. One possible explanation for this variability is increased exposure of certain populations to non-tuberculous mycobacteria (NTM). This study used a murine model to determine the effect that exposure to NTM after BCG vaccination had on the efficacy of BCG against aerosol *Mycobacterium tuberculosis* challenge. The effects of administering live *Mycobacterium avium* (MA) by an oral route and killed MA by a systemic route on BCG-induced protection were evaluated. CD4+ and CD8+ T cell responses were profiled to define the immunological mechanisms underlying any effect on BCG efficacy. BCG efficacy was enhanced by exposure to killed MA administered by a systemic route; T helper 1 and T helper 17 responses were associated with increased protection. BCG efficacy was reduced by exposure to live MA administered by the oral route; T helper 2 cells were associated with reduced protection. These findings demonstrate that exposure to NTM can induce opposite effects on BCG efficacy depending on route of exposure and viability of NTM. A reproducible model of NTM exposure would be valuable in the evaluation of novel TB vaccine candidates.

## 1 Introduction

Tuberculosis (TB) is caused by the bacterium *Mycobacterium tuberculosis* (*M. tb*). It was estimated that in 2011, 12 million people worldwide were suffering with TB disease and a further 8.7 million people are infected each year [1]. The number of people living with latent *M. tb* infection may be nearer 2 billion [2]. The incidence is greatest in Asia (59% of TB cases) and Africa (26%) and the burden of disease is particularly high in countries with high HIV prevalence [1].

Bacillus Calmette-Guerin (BCG), an attenuated strain of *Mycobacterium bovis*, is the only licensed vaccine for use against TB. BCG induces CD4+ T helper type 1 and CD8+ T cell responses, which play a protective role in the host's immune response against *M. tb* [3], [4], [5]. A meta-analysis of randomised controlled trials and case–control studies that evaluated the efficacy of BCG vaccination against pulmonary TB showed efficacy to vary between 0% and 80% across these trials [6], [7]. Forty one per cent of this variation could be attributed to the geographical location where the trial was conducted [6]. There are a number of theories to explain the variable efficacy of BCG vaccination against pulmonary TB. Animal studies have shown genetically different strains of BCG have varying levels of immunogenicity but the observation that the same vaccine preparation confers significantly different levels of protection in different human populations implies that BCG strain difference is probably not the only explanation [8], [9], [10], [11]. Certain strains of *M. tb* are more virulent than others and BCG vaccine efficacy is impaired in mice infected with these high-virulence strains [12], [13]. Helminth co-infection is associated with a reduction in the immunogenicity of BCG vaccination in a mouse model [14], [15], [16]. Exposure to non-tuberculous strains of mycobacteria (NTM) in the environment may also contribute to the variability of BCG efficacy [17], [18].

Currently, at least 55 species of NTM have been identified [19]. NTM are ubiquitous in the environment, more prevalent in hot climates than cold climates and are often associated with soil and water supplies [20], [21]. Around half of the species are capable of causing infection in humans and animals although these infections are generally opportunistic and often affect people with some degree of immune suppression [19]. In populations where BCG is less effective, there is a high level of exposure to NTM. This can be demonstrated by comparing, on a population level, the reactivity to purified protein derivatives (PPDs) of mycobacteria prior to BCG vaccination. The frequency of responders and the magnitude of interferon-γ (IFNγ) induced in response to PPDs of NTM species is far greater in Malawian and South Indian populations, where the efficacy of BCG against pulmonary TB is 0%, compared to the UK, where the efficacy of BCG is 80% [22], [23].

It has been shown in guinea pigs and mice that, like BCG, NTM can induce protective immunity against *M. tb* infection, although the level of protection is reported to be less than that conferred by BCG [24], [25], [26], [27], [28]. Detailed analyses of the immune responses induced by NTM infection are limited. An overview of reports indicates that IFNγ is commonly induced and production of TNFα, IL-1β and IL-6 has also been reported [5], [29], [30], [31]. There are also reports that NTM may induce mycobacteria-specific responses that are not protective against *M. tb* infection; for example a T helper 2 type response and the immune-modulatory cytokines IL-10 and TGFβ [31], [32], [33].

Several studies in animal models have assessed the effect of NTM exposure on the efficacy conferred by subsequent BCG vaccination against challenge with *M. tb* or *M. bovis*. Brandt et al. published data showing that subcutaneous infection of mice with a mix of *Mycobacterium avium*, *Mycobacterium vaccae* and *Mycobacterium kansasii*, which was subsequently cleared by antibiotic treatment prior to BCG vaccination, reduced the level of protection afforded against *M. tb* aerosol challenge [27]. A similar reduction of BCG efficacy was seen in guinea pigs when *M. avium* strain WAg206 was administered orally prior to BCG vaccination [24]. In a study of BCG efficacy in calves, no protection was conferred against *M. bovis* challenge in animals with PPD sensitivity prior to BCG vaccination [34]. This PPD sensitivity was attributed to natural exposure to NTM before recruitment of the calves to the study. In contrast to these studies, there are others that fail to show a reduction in BCG efficacy after exposure to NTM, highlighting the variability and inconsistency of these models of NTM exposure [25], [26], [35], [36], [37], [38].

To understand the effect of NTM exposure in populations where BCG fails to protect against pulmonary TB, we set out to model the pattern of NTM exposure that is likely to occur. Despite the variable efficacy of BCG against pulmonary TB, vaccination confers a high level of protection against childhood forms of severe TB (81%) and World Health Authority guidance is that in TB-endemic populations BCG should be administered at, or as soon after, birth as possible therefore exposure to NTM occurs after BCG vaccination [7], [19]. The route by which people are exposed to NTM is unknown. Exposure via the oral route in drinking water is a probable route, as is systemic exposure. This study evaluated two models of NTM exposure in mice previously vaccinated with BCG. The effect of NTM exposure on BCG vaccine efficacy was assessed by aerosol *M. tb* challenge and CD4+ and CD8+ T cell responses were profiled. We first modelled exposure to killed *M. avium* via the systemic route and found BCG efficacy was enhanced by this exposure. Flaherty et al. have previously published a model of oral exposure to *M. avium* after BCG vaccination and reported that exposure reduced the efficacy of BCG against *M. tb* challenge [39]. We replicated Flaherty's model to compare the immune responses induced in a model that compromises vaccine efficacy to the responses induced in a model that enhances vaccine efficacy with the view to determine the immunological mechanisms behind these differential effects on BCG-induced immunity.

## 2 Materials and methods

### 2.1 Ethics statement

Animal procedures were performed in accordance with the UK Animals (Scientific procedures) Act 1986 and were approved by the University of Oxford Animal Care and Ethical Review Committee (PPL 30/2414 and 30/2889).

### 2.2 Mycobacteria

BCG Pasteur (a kind donation from Dr A Rawkins) was grown in Middlebrook 7H9 media (with 10% ADC supplement and 0.05% Tween 80) at 37 °C to mid-log phase, aliquoted and frozen in PBS at −80 °C. *M. avium* (MA) strain 724, strain 2-151 (both a kind donation from Prof. I Orme) and MA strain 104 (a kind donation from Prof. A Cooper) were grown in supplemented Middlebrook 7H9 media to mid-log phase and then plated on Middlebrook 7H10 plates (with 10% oleic acid-albumen-dextrose-catalase (OADC) enrichment and 0.05% glycerol) for 2–3 weeks at 30 °C. Smooth-transparent and rough colonies were picked and suspended in 7H9 media, then incubated in a rotating incubator at 30 °C overnight to reduce clumping before freezing in PBS at −80 °C. MA 724 was chosen based on well documented use in NTM exposure models and it's ability to cause a persistent infection in mice, suggesting a degree of pathogenicity [25], [36]. MA 104 was the strain used by Flaherty et al. in the published study we replicated [39]. This strain was originally isolated from an AIDS patient and causes a persistent infection in mice [40]. *M. tb* Erdmann KO1 was supplied by the US Food and Drug Administration (FDA) or BEI Resources (Manassas Virginia, USA).

### 2.3 Mice and immunisations

Female C57BL/6 J mice were from Harlan Laboratories, UK. Animals were ordered at six weeks of age. Ex-breeders and nine week old mice were used as naïve controls in aerosol challenge experiments. Groups of animals were kept in a specific pathogen free facility within individually ventilated cages.

**Figure 1:**
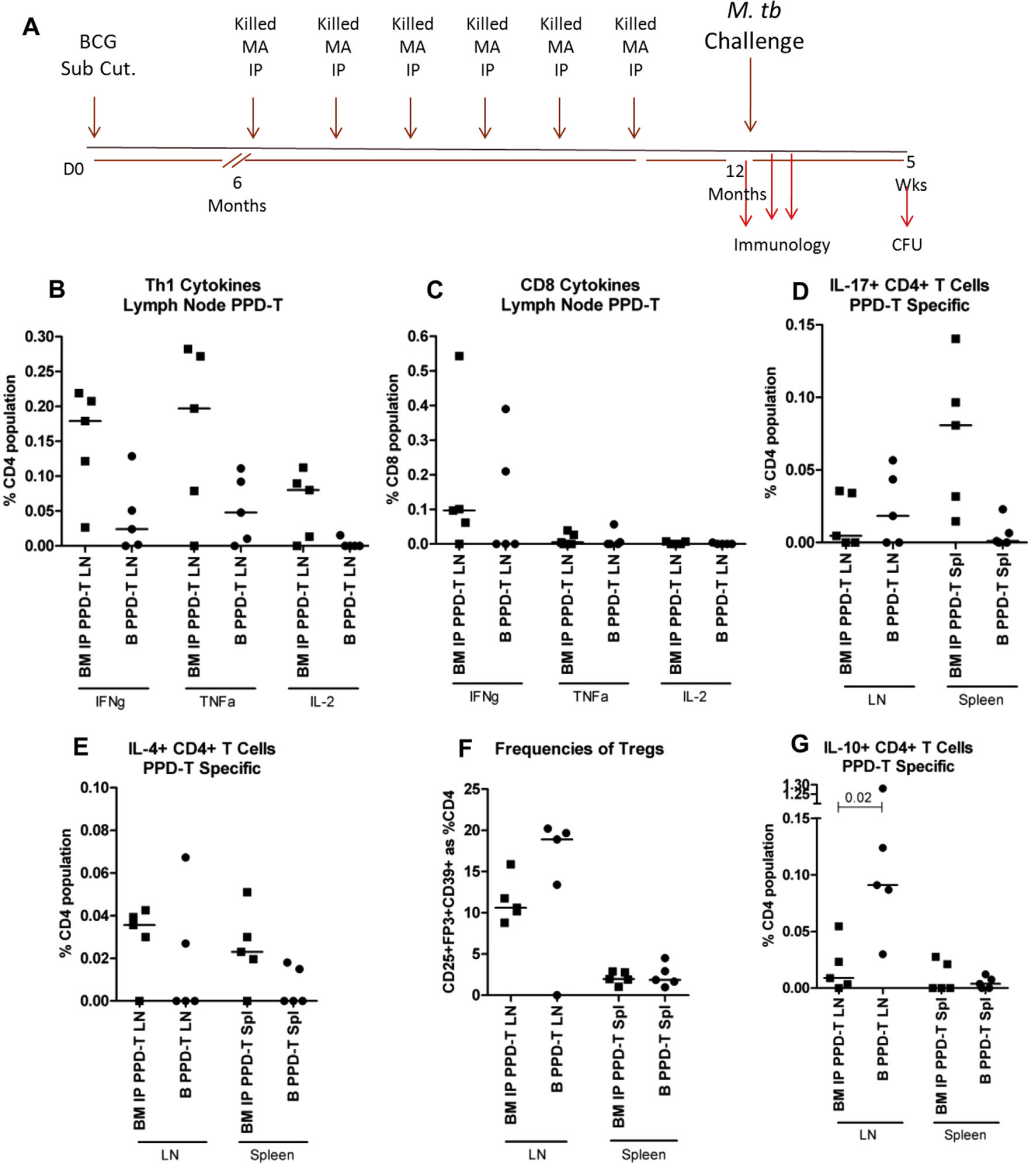
Cellular responses in BCG vaccinated mice that were or were not sensitised with MA 724 IP after BCG Responses were assayed at the 12 month time point; 4 weeks after the last dose of MA 724. Cells from inguinal lymph nodes (LN) and spleens were stimulated with PPD-T. (A) Experiment plan (B and C) IFNγ, TNFα and IL-2 production from CD4+ or CD8+ T cells in the LN, (D) IL-17 production from CD4+ T cells in the LN and spleen, (E) IL-4 production from CD4+ T cells in the LN and spleen, (F) expression of CD25, FoxP3 and CD39 on CD4+ T cells from the LN and spleen, (G) IL-10 production from CD4+ T cells in the LN and spleen. B = BCG vaccinated only, BM = BCG vaccinated mice which received MA 724. Results are expressed as a percentage of the CD4+ or CD8+ T cell population and are stimulation specific; media only control well values are subtracted. Each data point represents one mouse and the median is displayed, *n* = 5. The Mann–Whitney *U* test was used to determine statistical significance and is shown where differences were significant. Subcutaneous (Sub Cut.), Intraperitoneal (IP), *M. avium* (MA), Colony Forming Units (CFU), Day 0 (D0), Regulatory T cells (Tregs).

Frozen aliquots of bacteria were thawed and sonicated. Where MA was to be given heat-killed, the aliquots were placed in a water bath at 80 °C for 25 min. Bacteria were then diluted to the required concentration in sterile PBS before administration. 100 μl of BCG in PBS was injected subcutaneously (SC) at the base of the tail using a U-100 29G syringe. 1 × 10^5^ CFU was administered. 100 μl of MA 724 or 2-151 for intraperitoneal injection (IP) was suspended in PBS and administered with a U-100 29G syringe in the right or left side of the peritoneal cavity. In the IP exposure model 1 × 10^8^ CFU of killed MA strain 2-151 or 724 were administered. In the model of oral exposure mice received 100 μl of live MA 104 suspended in PBS via a dosing cannula inserted directly in to the stomach. 1 × 10^2^ CFU was administered. Mice were restrained but not anaesthetised for these procedures (Figure 1(A): Experiment plan of the IP exposure model; Figure 4(A): Experiment plan of the oral exposure model).

**Figure 2:**
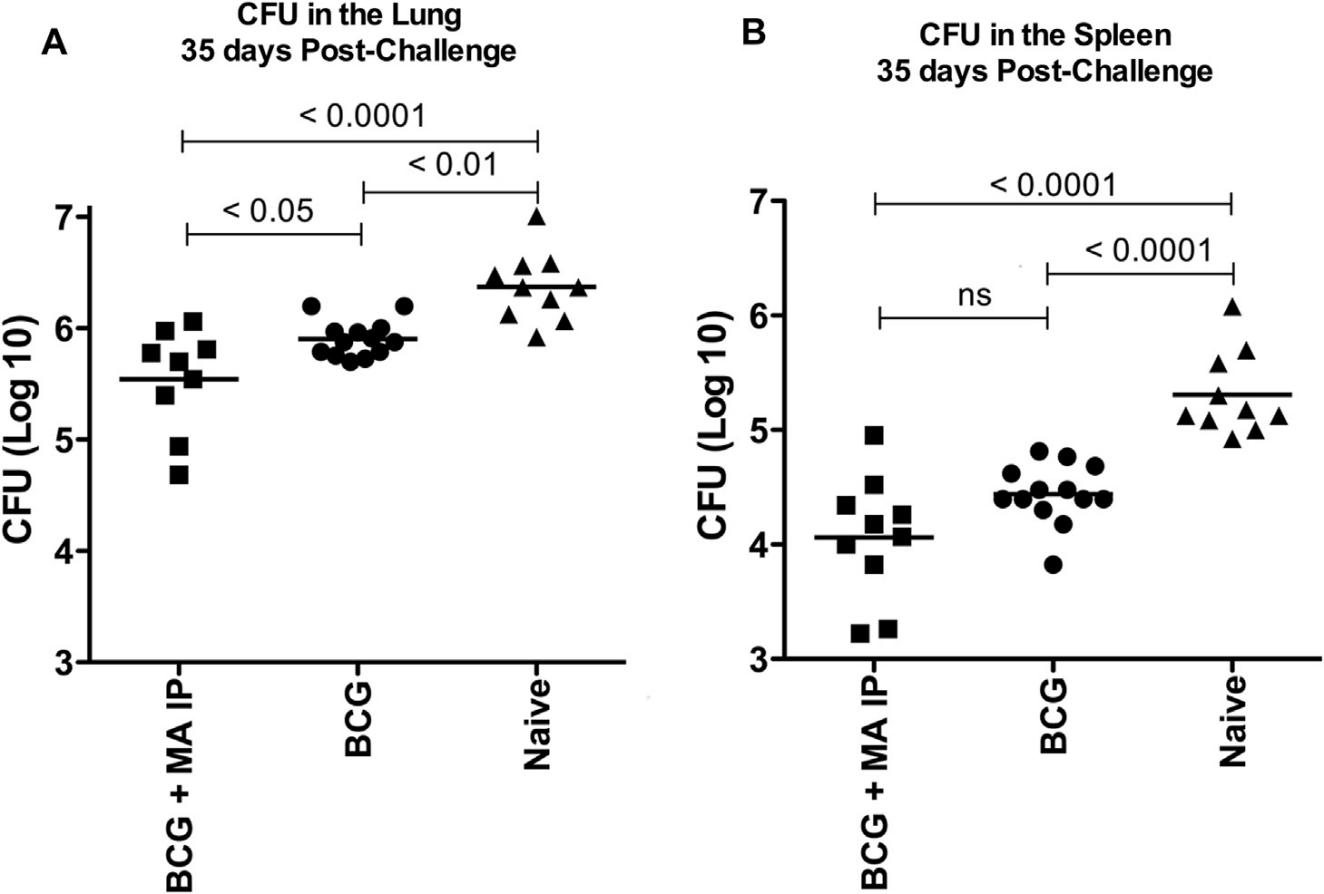
Burden of *M. tb* CFU in the lungs and spleens 35 days after challenge Groups included naïve, BCG vaccinated (BCG) and BCG followed by MA 724 IP exposure (BCG + MA IP). Data are Log (10) of the colony forming unit (CFU) count of the whole organ. Each data point represents one mouse and the mean is displayed. A One-way ANOVA with Bonferroni post test was used to determine statistical significance; the *p* value for each comparison is displayed.

**Figure 3:**
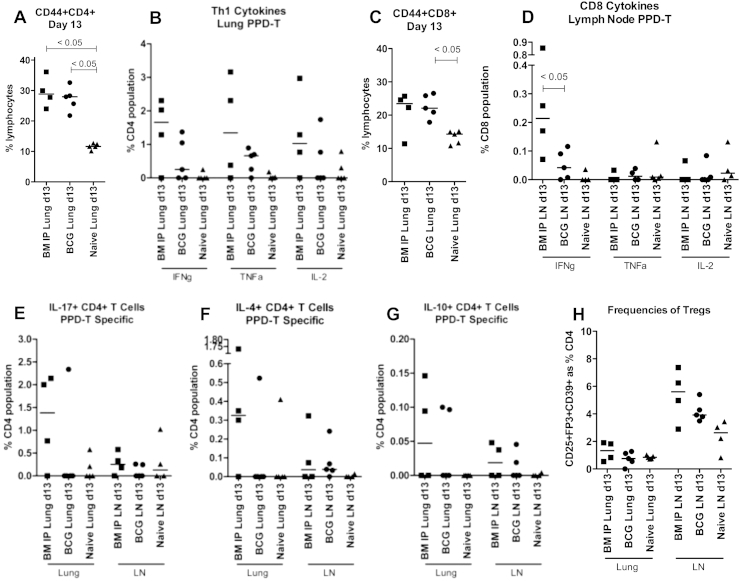
Cellular response to *M. tb* infection 13 days after challenge Thirteen and sixteen days after *M. tb* challenge cells were isolated from lungs and pulmonary LN (LN) of naïve mice, BCG-vaccinated mice (BCG) and mice vaccinated with BCG followed by MA 724 IP exposure (BM IP). Cells were stimulated with PPD-T and then stained for analysis by flow cytometry. (A) CD44 + CD4+ T cells in the lung, (B) IFNγ, TNFα and IL-2 production from CD4+ T cells in the lung, (C) CD44 + CD8+ T cells in the lung, (D) IFNγ, TNFα and IL-2 production from CD8+ T cells in the LN, (E) IL-17 + CD4+ T cells in the lung and LN, (F) IL-4+ CD4+ T cells in the lung and LN, (G) IL-10 + CD4+ T cells in the lung and LN, (H) expression of CD25, FoxP3 and CD39 on CD4+ T cells from the lung and LN. Cytokine responses are antigen-specific; media control values are subtracted. Regulatory T cell (Treg) frequency is not antigen-specific. Each data point represents one mouse and the median is displayed. The One-way ANOVA Kruskal–Wallis with Dunns post test was used to determine statistical significance; the *p* value is displayed where significance was calculated.

**Figure 4:**
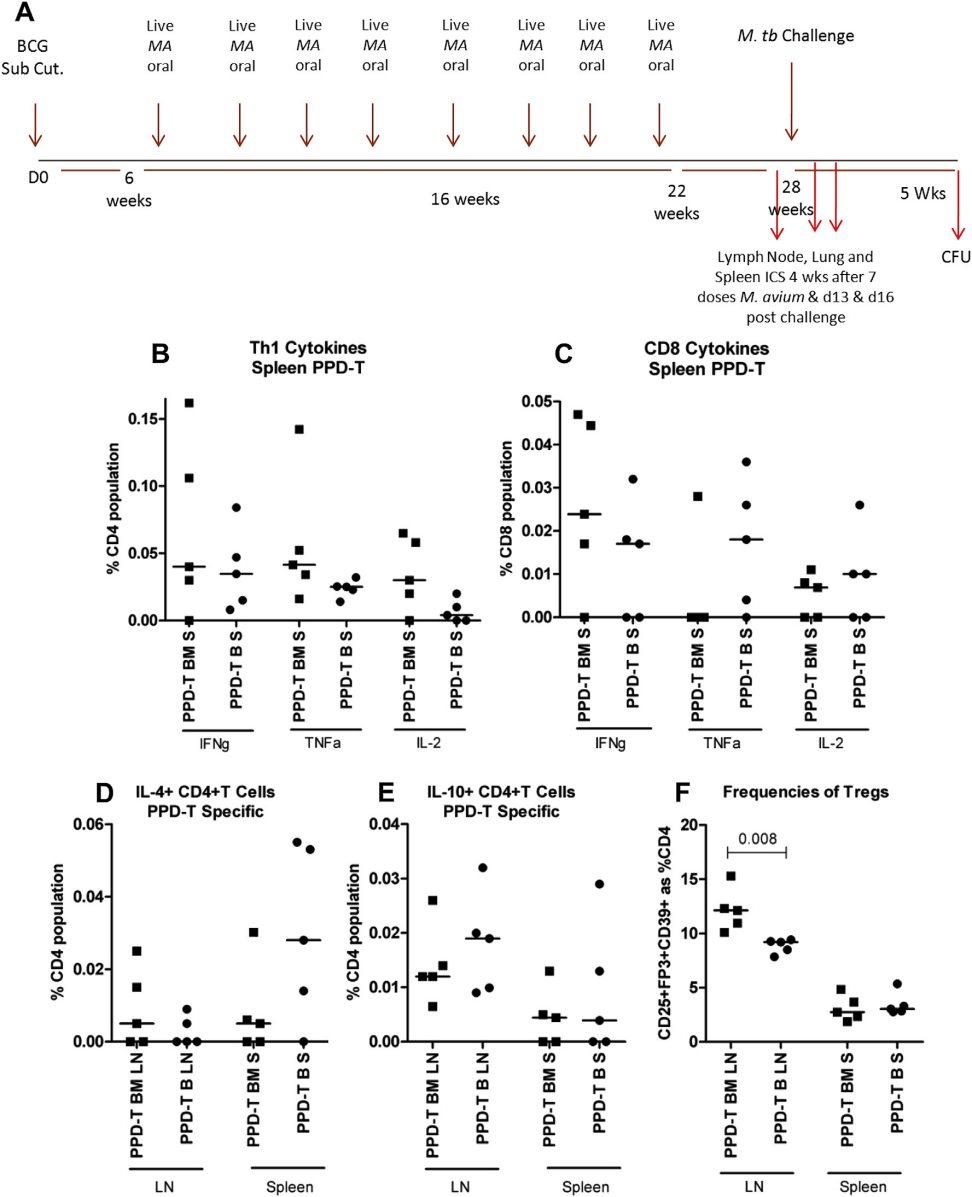
Responses induced by oral exposure to live MA 104 after BCG vaccination (A) Experimental plan; mice were orally exposed to live MA 104 8 times at 2 week intervals after BCG vaccination (BM) or left un-manipulated after BCG (B) and then challenged with *M. tb*. Immune responses were recorded at week 28, six weeks after the last dose of MA in the exposed group and directly before challenge with *M. tb*. Cells from mesenteric lymph nodes (LN) and spleens were stimulated with PPD-T then stained for analysis by flow cytometry. (B and C) IFNγ, TNFα and IL-2 production from CD4+ and CD8+ T cells in the spleen, (D) IL-4+ CD4+ T cells in the LN and spleen, (E) IL-10 + CD4+ T cells in the LN and spleen, (F) expression of CD25, FoxP3 and CD39 on CD4+ T cells from the LN and spleen. Cytokine responses are antigen-specific; media control values are subtracted. Regulatory T cell (Treg) frequency is not antigen-specific. Each data point represents one mouse and the median is displayed. The Mann–Whitney *U* test was used to test for statistical significance and is shown where differences were significant. Subcutaneous (Sub Cut.), *M. avium* (*MA*), Colony Forming Units (CFU), Day 0 (D0), Regulatory T cells (Tregs), Intracellular staining (ICS).

### 2.4 *M. tb* aerosol challenge

Mice were transferred to a containment level III (CL3) suite and loaded into nose-only restrainers. Restrainers were inserted into an exposure chamber inside a CL3 isolator. *M. tb* was diluted in sterile PBS and put in to a collision nebuliser. Dose escalation studies were performed to determine the concentration of *M. tb* needed to deliver 100 CFU to each mouse in a 10 min period of nebuliser activity followed by a five minute purge cycle with clean air. The Biaera aeroMP controller monitored the airflow and pressure in the apparatus. After exposure, mice were removed from the restrainers and housed in CL3 containment cages. For quantification of CFU in *M. tb* infected lungs or spleens the organs were homogenised in 1 ml of PBS using the Precellys^®^24 then plated on enriched Middlebrook 7H10 plates. After four weeks of incubation at 37 °C colonies were counted. Lungs from three naïve mice were taken 24 h after challenge to determine the dose of *M. tb* administered in the challenge. Vaccine efficacy was determined from CFU quantification on lung and spleen homogenates taken 35 days after challenge. In the model of oral exposure the burden of MA 104 CFU in the lungs and spleen was determined by also plating homogenates on Middlebrook 7H10 plates supplemented with 2 μg/ml Clarithromycin to exclude MA growth. There was no difference in the number of CFU between plates that contained antibiotic and those that did not, therefore we concluded there was no growth of MA 104 in the lungs or spleens of these mice.

### 2.5 Isolation and stimulation of cells

Mouse spleens and lymph nodes were mechanically homogenised in PBS, passed through a 70 μm cell strainer and erythrocytes in the spleens were removed by re-suspension of cells in ACK lysis buffer for five minutes. The reaction was stopped by PBS. Cells were centrifuged and resuspended in complete MEM (10% Foetal Calf Serum, Pen/Strep and l-glutamine). Lungs were perfused before dissection by injecting roughly 2 ml of sterile PBS into the heart. Lungs were then dissected into PBS, diced into 1 mm pieces and suspended in 5 ml of RPMI with 700 μg/ml collagenase and 30 μg/ml DNAse (Sigma) for 30 min at 37 °C. The reaction was stopped by adding complete MEM, then the suspension was passed through a 70 μm sieve and mashed with a 5 ml syringe. Samples were centrifuged and erythrocytes lysed with ACK lysis buffer. Cells were centrifuged and resuspended in complete MEM.

For flow cytometry, cells were incubated for six hours at 37 °C in the presence of PPD from *M. tb* (PPD-T) at 10 μg/ml. After two hours, 0.22 μl of GolgiPlug (Brefeldin A) was added. Following incubation, cells were stored at 4 °C overnight.

### 2.6 Multi-parameter flow cytometry

**Figure S1:**
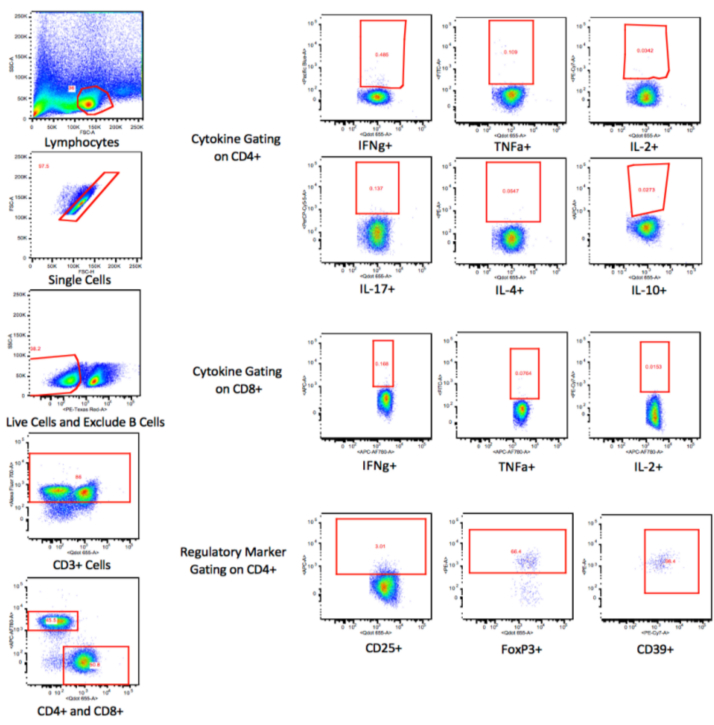
Representative flow cytometry data gating strategy Lymphocytes were selected on forward scatter area vs side scatter area then doublets and cell clumps excluded on forward scatter area vs height. Dead cells and B cells were excluded before selecting CD3+ cells. CD4+ T cells were selected from the CD8− population and vice versa for CD8+ T cells. Cytokines were gated on CD4+ or CD8+ T cells. For regulatory T cell phenotyping CD25 expression was gated on CD4+ T cells. On CD25 + cells FoxP3 expression was gated and then CD39 expression was gated on those cells.

Cells were centrifuged, washed with PBS and then stained with 10 μl of live/dead fixable stain diluted in PBS at the required concentration for 10 min. All incubations in this protocol were on ice and in the dark. Cell surface specific antibodies and FC block (anti CD16/32) were diluted in PBS with 2% FBS (PBS-FBS). Surface antibodies used in this study were B220 AF700 (clone RA3-6B2), B220 PE-Texas red (clone RA3-6B2), CD3 PerCP-Cy5.5 (clone 145-2c11), CD3 AF700 (clone 17A2), CD8a APC-ef780 (clone 53-6.7), CD4 eFluor 650NC (clone GK1.5), CD44 AF700 (clone IM7), CD25 APC (clone PC61.5) and CD39 PE-Cy7 (clone 24DMS1). The cocktail of surface antibodies was added to the live/dead stain and incubated for 30 min. If cells were to be stained for intracellular markers they were then washed in PBS-FBS and permeabilised by incubation with Cytofix/Cytoperm for 10 min. Cells were washed in PermWash and then incubated for 30 min with an intracellular staining cocktail and Fc block diluted in PermWash. Intracellular antibodies used in this study were IFNγ Brilliant Violet 421 (clone XMG1.2), IL-2 PE-Cy7 (clone JES6-5H4), IL-17a PerCP-Cy5.5 (clone eBio17B7), TNFα FITC (clone MP6-XT22), IL-4 PE (clone 11B11), IL-10 APC (clone JES5-16E3) and FoxP3 PE (clone FJK-16S). Cells were finally resuspended in PBS-FBS for analysis. For *M. tb* infected samples the protocol for cell staining was followed as above but at CL3 containment. After the final stage of staining the cells were fixed in PBS-FBS with 4% paraformaldehyde for 30 min. The samples were then washed and resuspended in PBS-FBS then transferred to a sterile 96 well U-bottom plate and removed from CL3. Samples were run on a BD LSRII cytometer linked to Facs Diva software and analysed with Flow Jo v8.8.7 and 9 (Tree Star Inc.). An example of typical gating strategies is shown in Supplementary Figure 1. For gating of cytokines the media control sample was used to determine where the negative population fell. For CD44 a preliminary experiment with fluorescence minus one (FMO) was performed to determine the level of marker expression. For some experiments SPICE was used to display complicated data comparisons.

### 2.7 Statistics

All data were graphed and analysed with Prism v5.0 (GraphPad Software). Flow cytometry data are presented as an antigen-specific response by subtracting media-only control values from the antigen-specific value prior to analysis. These data were non-parametric therefore the median value was calculated and significance between groups tested with the Mann–Whitney *U* test. Where comparison of multiple groups was made the One-way ANOVA Kruskal–Wallis with Dunns post-test was applied. CFU Log(10)data was tested for normal distribution using a D'Agostino and Pearson omnibus normality test. The mean was calculated for these samples and significance between groups tested with a one-way analysis of variance and Bonferroni post-test. Differences were considered statistically significant if *p* < 0.05.

## 3 Results

### 3.1 Exposure to killed MA IP after BCG vaccination increases the magnitude of Th1 and CD8+ T cells

**Figure S2:**
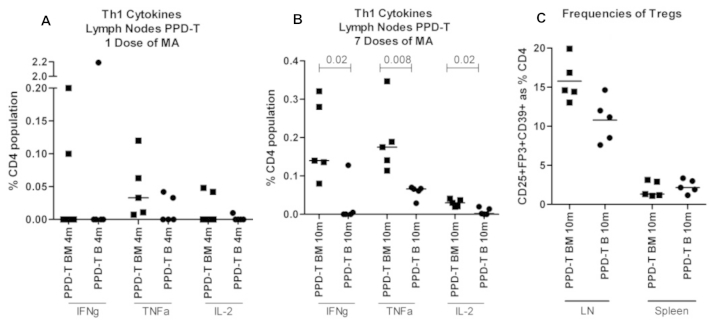
Th1 responses in BCG vaccinated mice and mice vaccinated then exposed to killed MA 2-151 IP Mice were vaccinated with BCG subcutaneously then 12 weeks later half the mice received killed MA 2-151 once a month for 7 months. Cells from inguinal lymph nodes and spleens were stimulated with PPD-T and then stained for analysis by flow cytometry. (A) IFNγ, TNFα and IL-2 production from CD4+ T cells in the LN 4 weeks after 1 dose of MA 2-151 was administered to the group receiving MA 2-151 after BCG vaccination; this was 4 months after BCG was administered to both groups, (B) IFNγ, TNFα and IL-2 production from CD4+ T cells in the LN 4 weeks after 7 doses of MA; this was 10 months after BCG, (C) expression of CD25, FoxP3 and CD39 on CD4+ T cells from the LN and spleen. Results are expressed as a percentage of the CD4+ T cell population. BCG with MA 2-151 exposure (BM), BCG alone (B). Cytokine responses are antigen-specific; media control well values are subtracted. Regulatory T cell (Treg) frequency is not antigen-specific. Each data point represents one mouse and the median is displayed. The Mann–Whitney *U* test was used to test for statistical significance and is shown where differences were significant. 4 months since BCG (4m), 10 months since BCG (10m).

We set out to model human NTM exposure in order to assess it's effect on the immune response induced by BCG. We reasoned that human exposure is likely to be repeated exposure to NTM at systemic surfaces; such exposures do not generally cause pathological infections in immunocompetent people. Mice were vaccinated with BCG then given MA intraperitoneally (IP) once a month for 6 months. Mice are susceptible to pathological infection from this strain of MA therefore MA was administered killed to avoid the induction of a pathological infection. From a previous experiment we knew that giving repeated doses of killed MA to BCG vaccinated mice produced a stronger immune response than a single dose of killed MA did (Supplementary Figure 2). An aerosol *M. tb* challenge was conducted to assess the effect of MA exposure on the protection conferred by BCG vaccination. Immune responses were characterised prior to *M. tb* challenge and 13 and 16 days after challenge. The experiment plan is summarised in (Figure 1(A)).

**Figure S3:**
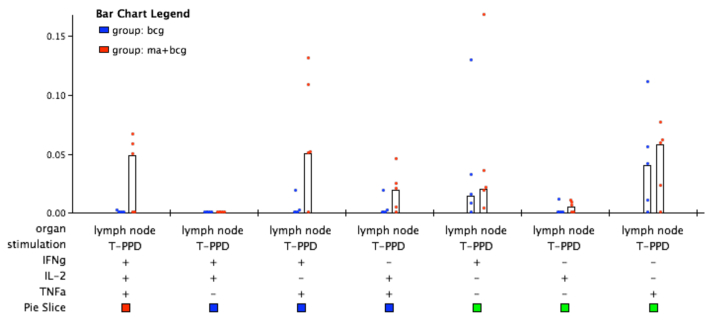
Cellular responses in BCG vaccinated mice that were or were not sensitised with MA 724 IP after BCG Responses were assayed at 12 months; 4 weeks after the last dose of MA. Cells from inguinal lymph nodes were stimulated with PPD-T. The secretion of one or more of IFNγ, TNFα and IL-2 from CD4+ T cells is shown. Results are expressed as a percentage of the CD4+ T cell population and are antigen-specific; media only control well values are subtracted. Each data point represents one mouse and the median is displayed, *n* = 5. PPD-T (T-PPD).

Prior to challenge, in the inguinal lymph node (iLN) of mice that received BCG alone PPD-T specific IFNγ+ CD4+ T cells and TNFα+ CD4+ T cells were recorded at a frequency of 0.03% and 0.05% respectively (Figure 1(B)). Exposure to 6 doses of killed MA 724 after BCG vaccination induced IFNγ+ and TNFα+ PPD-specific CD4+ T cells at 0.2% as well as IL-2+ CD4+ T cells at 0.1%. These responses were greater compared to the BCG alone mice, however the difference was not statistically significant. The Th1 response was more polyfunctional after exposure to MA; with greater proportions of PPD-T specific CD4+ T cells secreting IFNγ, TNFα and IL-2, and CD4+ T cells secreting both IFNγ and TNFα compared to the BCG alone group (Supplementary Figure 3 ). No PPD-T specific CD8+ T cell responses were detected in mice vaccinated with BCG alone whereas a PPD-T specific IFNγ+ CD8+ T cell response was detected in MA-exposed group (Figure 1(C)). Similar frequencies of IL-17 + CD4+ T cells were detected between the groups in the iLN (Figure 1(D)). IL-17 + CD4+ T cells were recorded in the spleen of the MA-exposed group whereas the median frequency was 0% in the BCG alone group. In the MA-exposed group the frequency of IL-4+ CD4+ T cells in the iLN and spleen were 0.04% and 0.02% respectively, in the BCG alone group it was 0% in both organs (Figure 1(E)). The frequency of regulatory T cells (Tregs) in the iLN of the MA-exposed group was 10% and in the BCG alone group 19%, this difference was not statistically significant (Figure 1(F)). The frequency of Tregs in the spleen of both groups was comparable. The median frequency of PPD-T specific IL-10 + CD4+ T cells was 0.01% in the iLN of the MA-exposed group and 0.09% in the BCG alone group; this difference was significant (*p* = 0.02; Figure 1(G)). IL-10 responses in the spleen were very low in both groups.

### 3.2 Exposure to killed MA IP after BCG vaccination increases protection against *M. tb* challenge

Thirty-five days after aerosol *M. tb* challenge, the group receiving MA 724 after BCG had a significantly lower CFU burden in the lungs compared to the BCG alone group (*p* = 0.017 lungs) (Figure 2). The mean CFU count in the spleen of the group that received MA 724 after BCG was lower compared to the group that received BCG alone but this difference was not significant. The mean Log(10) CFU count in the naïve group was 6.37 lung; 5.31 spleen, in the BCG alone group 5.9 lung; 4.44 spleen and in the group that received MA 724 after BCG 5.54 lung; 4.1 spleen. Both the BCG alone group and the group that received MA 724 after BCG had a significantly lower CFU burden in the lungs and spleen compared to naïve mice (*p* = 0.0001 lungs, *p* = <0.0001 spleen BCG alone; *p* = 0.0002 lungs, *p* = <0.0001 spleen BCG + MA).

### 3.3 Exposure to killed MA IP after BCG vaccination enhances Th1, Th17 and CD8+ T cell responses in the lung during *M. tb* infection

Thirteen and 16 days after challenge with *M. tb*, the cellular immune responses in the lungs, pulmonary lymph nodes (pLN) and spleens were assayed. Day 16 data were similar to day 13 data, so only day 13 data are presented. CD44 expression was used as a marker for CD4+ and CD8+ T cell activation. Thirteen days after *M. tb* challenge, there were significantly greater frequencies of activated CD4+ T cells in the lungs of the BCG alone and the MA-exposed groups compared to naïve mice (*p* < 0.05B + M and BCG alone; Figure 3(A)). The same was also seen in the pulmonary LN (pLN) and the spleen (*p* < 0.05B + M and BCG alone pLN; *p* < 0.05B + M and BCG alone spleen; data not shown). The median frequency of IFNγ+, TNFα+ and IL-2+ CD4+ T cells in the lungs of the MA-exposed group was 1–1.5%. In the BCG alone group the median frequency of IFNγ+ CD4+ T cells was 0.2% and TNFα+ CD4+ T cells was 0.8%. This was a lower frequency compared to the MA-exposed group although not statistically significant (Figure 3(B)). No Th1 response was detected in the lungs of the naïve group at day 13 or day 16.

The frequencies of activated CD8+ T cells were significantly higher in the lungs of the BCG alone group compared to naïve mice (*p* < 0.05 BCG alone lungs; Figure 3(C)). Frequencies of activated CD8+ T cells were significantly higher in the pLN of the MA-exposed group compared to naïve mice (*p* < 0.05B + M pLN; data not shown). In the pLN at day 13 the frequency of IFNγ+ CD8+ T cells in the MA-exposed group was significantly higher than in the BCG alone group (0.2% compared to 0.03%, *p* < 0.05; Figure 3(D)). No IFNγ+ CD8+ T cells were detected in the pLN of naïve mice. Frequencies of TNFα+ and IL-2+ CD8+ T cells in the pLN were low in all groups.

IL-17 + CD4+ T cells were detected 13 days after *M. tb* challenge in the lung of the MA-exposed group but not the BCG alone or naïve groups; the median frequency was 1.5% (Figure 3(E)). IL-4+ CD4+ T cells were also detected in the lungs of the MA-exposed group but not the BCG alone or naïve groups; the median frequency was 0.3% (Figure 3(F)). A low level of IL-10 + CD4+ T cells were detected in the lungs and pLN of the MA-exposed group and the BCG alone group; 0.05% and 0.03% respectively (Figure 3(G)). Frequencies of regulatory T cells in the lungs of all groups were around 1%. The frequency in the pLN of the MA-exposed group was 6%, the BCG alone group was 4% and the naïve group 3%; these differences were not statistically significant.

### 3.4 Exposure to live MA orally after BCG vaccination enhances Treg frequencies in the mesenteric lymph nodes

We replicated a previously published model where live MA is administered orally after BCG vaccination [39]. Repeated doses of live MA strain 104 (MA 104) were administered to mice at a low dose of 100 CFU to induce a sub-clinical infection. Analysis of CD4+ and CD8+ T cell responses were performed before and after challenge with *M. tb*: the model is represented in Figure 4(A).

After eight doses of live MA 104, the frequencies of PPD-T specific IFNγ+ and TNFα+ CD4+ T cells in the spleen were similar between the BCG alone and BCG – oral MA groups; median frequency 0.03–0.04% (Figure 4(B)). The BCG – oral MA group had a greater frequency of IL-2+ CD4+ T cells compared to the BCG alone group. PPD-T-specific IFNγ+, TNFα+ and IL-2+ CD8+ T cells were detected in the spleen of the BCG alone group; IFNγ+ and IL-2+ CD8+ T cells were detected in the BCG – oral MA group (Figure 4C). Frequencies of IFNγ+ and IL-2+ CD8+ T cells were similar between the groups at 0.02% and 0.01% respectively. Frequencies of IL-4+ CD4+ T cells specific for PPD-T were measured in the mesenteric LN (mLN) of the BCG – oral MA group at around 0.01%, a median frequency of 0% was detected in the BCG alone group (Figure 4D). PPD-T specific IL-4+ CD4+ T cells were detected in the spleens of the BCG – oral MA and BCG alone groups at 0.01% and 0.03% respectively; the difference was not statistically significant. PPD-T-specific IL-10 + CD4+ T cells were detected in the mLN of the BCG – oral MA group and the BCG alone group at 0.01% and 0.02% respectively (Figure 4(E)). The frequency of IL-10 + CD4+ T cells in the spleen was comparable between the groups. Frequencies of regulatory T cells in the mLN were significantly greater in the BCG – oral MA group compared to the BCG alone group; 13% and 9% respectively (*p* = 0.008; Figure 4(F)). Both groups had a median frequency of regulatory T cells around 3% in the spleen.

### 3.5 Oral exposure to live MA after BCG vaccination reduces protection against *M. tb*

**Figure 5:**
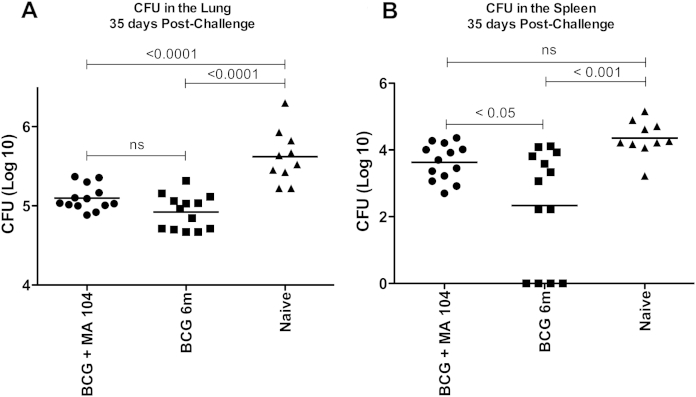
Burden of *M. tb* CFU in the lungs and spleens 35 days after challenge Groups in the challenge included naïve mice, BCG vaccinated mice (BCG) and BCG vaccinated followed by oral MA 104 exposure mice (BCG + MA 104). Data is Log (10) of the CFU count of the whole organ. Each data point represents one mouse and the mean is displayed. A One-way ANOVA with Bonferroni post test was used to determine statistical significance; the *p* value for each comparison is displayed. Colony forming units (CFU), 6 months since BCG (6 m).

Thirty five days after aerosol *M. tb* challenge, the group that received MA 104 orally after BCG had a significantly greater CFU burden in the spleen compared to the BCG alone group (*p* = <0.05 spleen). The mean CFU count in the lung of the group receiving MA 104 orally after BCG was greater compared to the group that received BCG alone but this difference did not reach statistical significance. The mean Log(10) CFU counts in the naïve group was 5.62 lung; 4.36 spleen, in the BCG alone group 4.92 lung; 2.34 spleen and in the group that received MA 104 after BCG 5.1 lung; 3.63 spleen (Figure 5). Both the BCG alone group and the group receiving MA 104 orally after BCG had a significantly lower CFU burden in the lungs compared to naïve mice and the BCG alone group had significantly lower CFU burden in the spleen compared to naïve mice (*p* = <0.0001 lungs, *p* = <0.001 spleen BCG alone; *p* = <0.0001 lungs, *p* = ns spleen BCG – oral MA;Figure 5(A) and (B). The CFU burden in the spleen of the group receiving MA 104 orally after BCG was not significantly different to the naïve group.

### 3.6 The cellular immune response during *M. tb* infection is altered by oral exposure to MA after BCG vaccination

**Figure 6:**
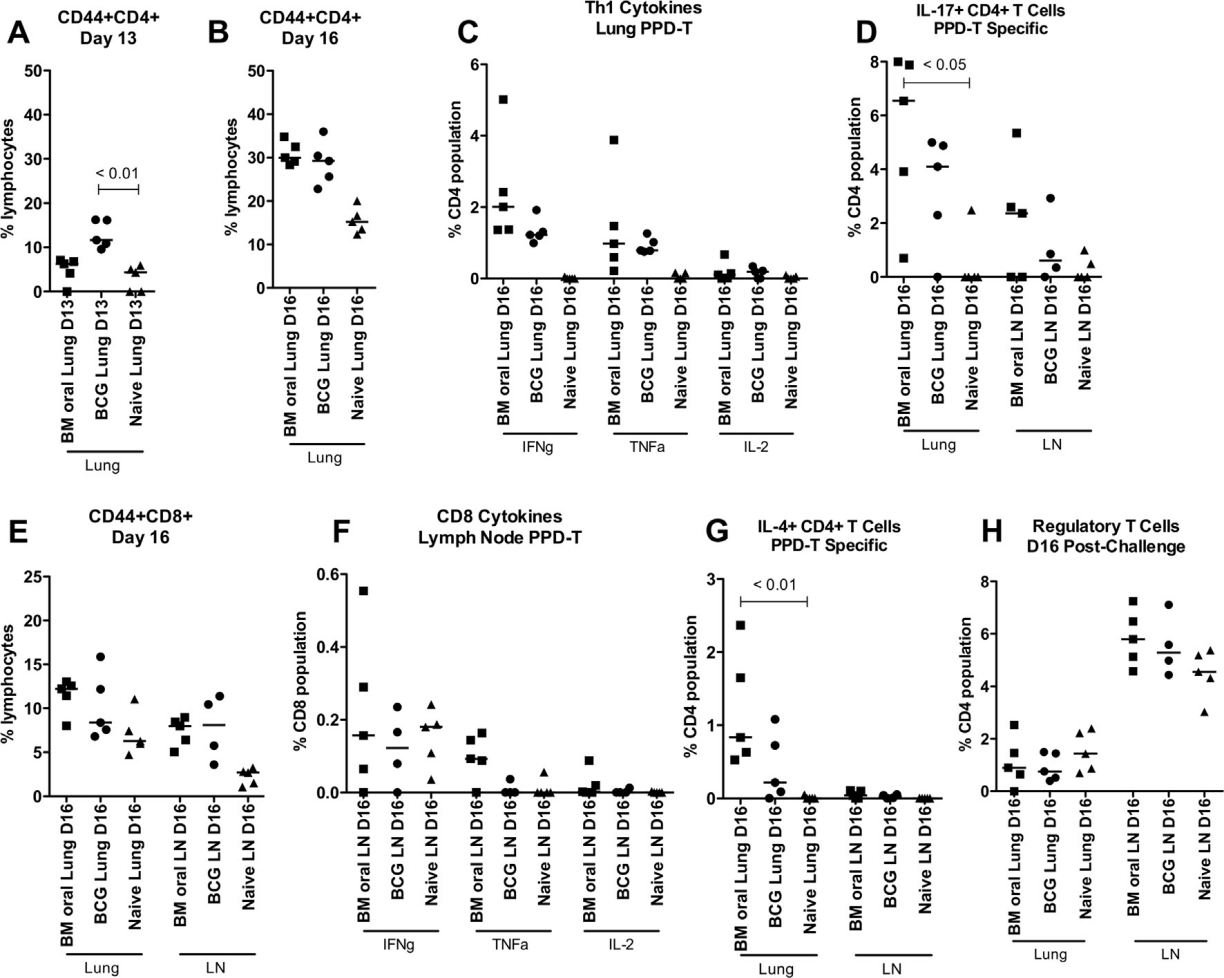
Cellular response to *M. tb* infection 16 days after challenge Thirteen and sixteen days after *M. tb* challenge cells were isolated from lungs and pulmonary lymph nodes (LN) of naïve mice, BCG vaccinated mice (BCG) and BCG vaccinated mice followed by oral MA 104 exposure (BM oral). Cells were stimulated with PPD-T and then stained for analysis by flow cytometry. (A and B) CD44 + CD4+ T cells in the lung at day 13 and 16 after infection, (C) IFNγ, TNFα and IL-2 production from CD4+ T cells in the lung at day 16, (D) IL-17 + CD4+ T cells in the lung and LN at day 16, (E) CD44 + CD8+ T cells in the lung and LN at day 16, (F) IFNγ, TNFα and IL-2 production from CD8+ T cells in the LN at day 16, (G) IL-4+ CD4+ T cells in the lung and LN at day 16, (H) expression of CD25, FoxP3 and CD39 on CD4+ T cells from the lung and LN at day 16. Cytokine responses are antigen-specific; media control well values are subtracted. Regulatory T cell frequency is not antigen-specific. Each data point represents one mouse and the median is displayed. The One-way ANOVA Kruskal–Wallis with Dunns post test was used to determine statistical significance; the *p* value is displayed where significance was calculated.

We also measured the responses of lymphocytes following *M. tb* challenge in the oral exposure model. Again, CD44 expression was used as a marker for CD4+ and CD8+ T cell activation. Thirteen days after challenge, significantly greater frequencies of activated CD4+ T cells were detected in the lungs of the BCG alone group compared to the naïve group; 12%–8% respectively (*p* < 0.01; Figure 6(A)). 16 days after challenge the frequency of activated CD4+ T cells in the lungs was similar between the BCG alone and BCG – oral MA groups at 30%; this was greater than the frequency in the naïve group which was 15%, although the difference did not reach statistical significance (Figure 6(B)). The same trend was also seen in the pLN and spleen at day 16 (data not shown). No PPD-T-specific cytokine responses were detected at day 13 in any of the groups (data not shown). At day 16 IFNγ+ and TNFα+ CD4+ T cells were detected in the lungs of the BCG alone and the BCG – oral MA groups at similar frequencies; 1.5–2% (Figure 6(C)). Median frequencies of activated CD8+ T cells were greater in the lungs, pLN and spleen of the BCG alone and BCG – oral MA groups compared to the naïve group; however these differences were not significant (Figure 6(E); spleen not shown). The frequency of IFNγ+ CD8+ T cells in the pLN was similar between all 3 groups; median frequencies 0.18–0.2% (Figure 6(F)). TNFα+ CD8+ T cells were detected in the BCG – oral MA group but not the BCG alone or naïve groups. Only a low level CD8+ T cell response was detected in the lungs at this time (data not shown).

A high frequency of IL-17 + CD4+ T cells was detected in the lungs of the BCG – oral MA group; 6.5% (Figure 6(D)). In the lungs of the BCG alone group 4% of CD4+ T cells were IL-17 +; this was lower than recorded in the BCG – oral MA group however the difference was not statistically significant. A similar trend was seen in the pLN but at lower frequencies. A greater frequency of IL-4+ CD4+ T cells was detected in the lungs of the BCG – oral MA group compared to the BCG alone group; 0.8% and 0.2% respectively; the difference was not statistically significant between these groups but was against naive mice (*p* < 0.01; Figure 6(G)). No IL-4+ CD4+ T cell response was recorded in the pLN. Regulatory T cell frequencies were similar between the 3 groups in the lungs (Figure 6(H)). In the pLN a similar frequency was recorded in the BCG – oral MA group and BCG alone groups at around 5.5%. Fewer regulatory T cells were recorded in the naïve group; this difference was not significant. There was no difference in the IL-10 response between the groups (data not shown).

## 4 Discussion

Thirty-five days after *M. tb* challenge, mice exposed to killed MA-IP after BCG vaccination had a significantly lower CFU burden in lungs compared to mice receiving BCG alone, demonstrating that exposure to killed MA by the IP route after BCG can increase efficacy of BCG vaccination. Early infiltration of T helper 1 and T helper 17 responses into the lungs during *M. tb* infection were associated with increased protection. Mice that had been exposed to live MA orally after BCG had a higher burden of *M. tb* in the lungs and spleens compared to mice that received BCG alone, demonstrating that exposure to live MA by the oral route after BCG reduced the efficacy of BCG vaccination. Th2 responses in the lungs during *M. tb* infection were associated with reduced protection. There have been previous reports of NTM exposure enhancing immunogenicity and protection against *M. tb* challenge in comparison to BCG alone; Edwards et al. showed protection was enhanced by *Mycobacterium intracellulare* exposure after BCG vaccination in a guinea pig study and Howard et al. reported enhanced immunogenicity in calves sensitised with MA prior to BCG vaccination compared to calves which received only BCG [29], [38]. The observation that oral exposure to live MA reduced the efficacy of BCG vaccination is in line with data reported by Flaherty et al. (in the study from which the model was replicated) [39]. Others have reported similar reductive effects on BCG efficacy in animal models, as discussed previously [24], [27], [34].

At day 13 after *M. tb* infection mice that had been exposed to killed MA-IP after BCG had higher frequencies of Th1 and Th17 subsets in the lungs compared to the BCG alone mice. Prior to challenge it was observed that repeated doses of killed MA-IP had increased the magnitude of IFNγ, TNFα and IL-2 producing CD4+ T cells and the ability of these cells to produce more than one of these cytokines. A polyfunctional Th1 profile has been linked to improved vaccine efficacy against *M. tb* in mice [41]. The enhanced Th17 and Th1 responses seen in the lungs of the killed MA-exposed mice during *M. tb* infection was likely due to the enhanced frequencies of these subsets observed pre-infection. This may have contributed to the increased protection observed in this group compared to the BCG alone mice; an early Th17 response in the lung has been shown to aid protection by induction of chemokines in the lungs that promote chemotaxis of Th1 cells to this site early in infection [42]. Prior to *M. tb* infection we observed a CD8+ T cell response in the killed-MA exposed mice but not in the BCG alone mice, this may have been due to a wane in the response in the BCG alone mice given vaccination was 12 months previously and they received no further mycobacterial exposure. Frequencies of CD8+ T cells were significantly greater in the LN of the killed MA-exposed mice 13 days after infection compared to the BCG alone mice and we also observed a greater frequency in the lungs at day 16 after infection. Mittrucker et al. correlated an early accumulation of CD8+ T cells to vaccine-mediated protection in *M. tb* infection; this study suggests that the enhanced frequency of this subset in our model may have played a role in controlling *M. tb* [43].

Unlike the killed IP model, we did not see an enhanced Th1 response before *M. tb* infection in mice exposed to live MA-orally compared to the BCG alone mice. Instead, we observed a significant increase in the frequency of Tregs in the mesenteric lymph nodes. The intestinal immune system is highly specialised to deal with constant antigen exposure from intestinal flora and food antigens and induces a tolerising environment to prevent inflammation against these common antigens [44], [45]. Studies of oral MA infection have reported low levels of pro-inflammatory cytokines and instead a local and systemic IL-10 response induced during infection [46], [47]. Furthermore, *in vitro* analysis has shown that intestinal macrophages infected with MA secreted lower levels of TNFα compared to their systemic counterparts; blocking IL-10 and TGFβ allowed these macrophages to kill intracellular bacteria [48]. During *M. tb* infection we observed a delayed infiltration of activated CD4+ T cells to the lungs of the mice that received oral MA after BCG compared to mice that received BCG alone. It is possible the delayed CD4+ T cell infiltration in the live MA-oral model may be linked to the reduction in vaccine efficacy, this is in agreement with the association of an early Th1 and Th17 pulmonary response and increased protection that we observed in the killed MA-IP model. It is important to note that no cytokine production from CD4+ T cells was detected in the lungs of the BCG alone group at day 13, as a result the subset of CD44 + CD4+ T cells could not be defined and their functional capability at this time point remains questionable. Prior to *M. tb* challenge we observed a significantly enhanced Treg response in the mesenteric LN of the live MA exposed group, however this group had a similar frequency of Tregs in the pulmonary LN during *M. tb* infection compared to the BCG alone group. Given the similar frequency of Tregs between the groups during infection it is unlikely that regulatory responses were accountable for the delay in activated T cells accumulating in the lungs of MA-exposed mice during *M. tb* infection. By day 16 after infection both vaccinated groups had comparable frequencies of Th1 in the lungs and a similar response in all three groups was detected in the LN. A significantly greater frequency of Th17 cells was detected in the lungs of the MA-exposed group. These results show that by day 16 Th1 and Th17 immune responses were equivalent if not enhanced in the live MA-exposed group and suggests that the mechanism by which vaccine efficacy is reduced is not likely to be through a reduction of these cell subsets.

The appearance of Th2 immune responses during *M. tb* infection in the lungs of mice exposed orally to live MA is potentially important in terms of understanding the mechanisms behind the varying effect of NTM exposure on BCG efficacy. Th2 responses were also detected in mice exposed to killed MA-IP but the frequency of IL-4+ CD4+ T cells in killed MA-IP mice was lower compared to the orally exposed mice. It may be that the increased frequencies of IL-4+ CD4+ T cells in the lungs of the live MA-oral mice during *M. tb* infection has a negative impact on infection control. IL-4 can inhibit inducible nitrous oxide synthase activity, which is a key mechanism in the control of *M. tb* in macrophages [49]. IL-4 knockout mice were shown not to be better at controlling *M. tb* infection compared to C57BL/6 wild type controls, which suggests that a normal level of Th2 response is not detrimental in *M. tb* infection [50]. However, BALB/C mice mount a Th2 skewed immune response and are more susceptible to *M. tb* than C57BL/6 mice [51]. IL-4 is produced in the late stages of infection in BALB/C mice and is linked to an increase in pathology in this strain [51], [52]. These reports may imply that a higher ratio of Th2/Th1 in long-term infection is pathological. In the live MA-oral exposure model it is possible that the Th2 response may have begun to expand by day 35 after infection, which is why there was a moderate effect on the level of BCG efficacy at this time point. In the report published by Flaherty et al., *M. tb* infection was followed-up to day 120 and showed lower BCG efficacy in the MA-exposed group at this time point compared to the 35 day time point [39]. It may be that at this later stage Th2 responses have further expanded in the MA-exposed group compared to the BCG alone group. This hypothesis remains to be tested but is a plausible explanation for the reduced efficacy observed in mice exposed to live MA-orally after BCG. Functional studies could help delineate the importance of the Th2 response in lowering BCG mediated protection in the oral model. Further studies are also required to demonstrate whether the increased Th1 and Th17 infiltrate observed in the killed MA-IP model is the mechanism by which efficacy is enhanced in this model.

**Figure S4:**
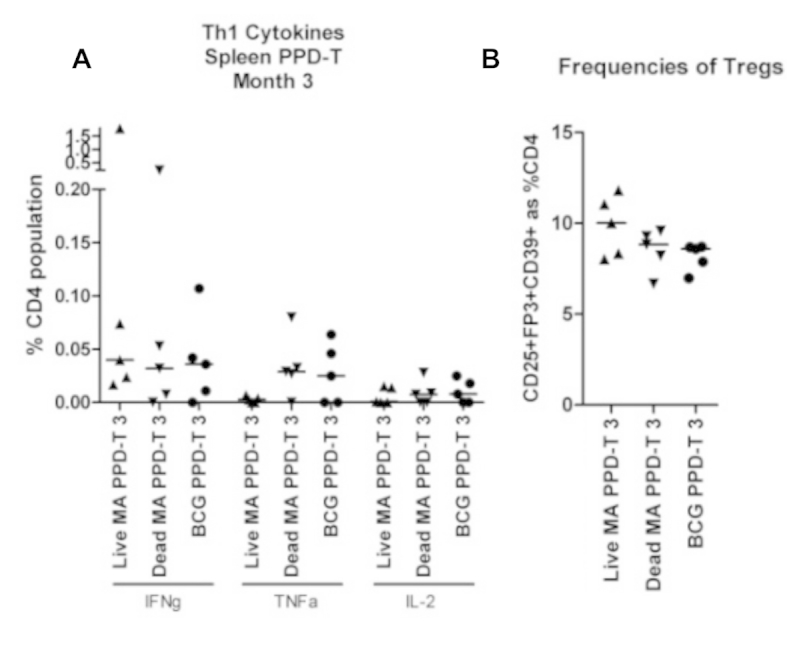
Cellular responses in mice infected with BCG, MA dead or MA live Mice were injected IP with either 1 × 10^5^ CFU of live MA or 1 × 10^8^ CFU of dead MA or SC with 1 × 10^5^ CFU of BCG. Responses were assayed at 3 months after infection. Splenocytes were stimulated with PPD-T. (A) IFNγ, TNFα and IL-2 production from CD4+ T cells, (B) expression of CD25, FoxP3 and CD39 on CD4+ T cells. Results are expressed as a percentage of the CD4+ T cell population. Cytokine responses are antigen-specific; media only control well values are subtracted. Each data point represents one mouse and the median is displayed, *n* = 5. PPD-T (T-PPD). The One-way ANOVA Kruskal–Wallis with Dunns post test was used to determine statistical significance; no significant differences were calculated.

Our study has focussed on comparing the immune response induced in a model of NTM exposure that enhances BCG efficacy to that induced in a model of NTM exposure that compromises BCG efficacy with the view to understand the immunological mechanisms that mediate these effects. The main limitation in our study design is that the models were not evaluated with the same strain of MA and that both live and dead bacteria were not tested in the same model. This has prevented us from delineating which parameters mediate the differential effects on vaccine efficacy; whether it is viability of the bacteria, the route of exposure or the strain of MA employed. However, the results have demonstrated that a model of NTM exposure that induces Th2 type responses and a regulatory response is associated with reduced vaccine efficacy. Further work can lead to determining which parameters are most important in inducing these types of responses and reduced vaccine efficacy. Live MA 724 could be assessed in the systemic model to evaluate the importance of MA viability in enhancing protective immunity. In previous studies the immune response induced by administering live MA 724 by the IP route was compared to the killed preparation. We found the Th1 and Treg response to be similar between the two, which suggests that viability may not affect the ability of MA exposure to enhance BCG efficacy via the IP route (Supplementary Figure 4). The administration of live MA orally induced a large Treg response, which was not seen when live MA was given IP. We conclude that the oral route of exposure induces this regulatory phenotype implicating route as a contributing factor to the reduced efficacy observed in the MA-oral model. These experiments are not directly comparable as no prior BCG vaccination was given when live MA IP was tested, although we do not think prior BCG would have changed the immune response we measured. Whether or not viability is important in reducing vaccine efficacy in the MA-oral model is an important further experiment. We suspect MA may need to be living to exert these immunological effects. Sangari et al. report that MA 104 invades the intestinal mucosa through enterocytes and enables it to disseminate and infect intestinal macrophages, which is likely to be an important step in modulating the immune response against the mycobacterium [53]. Both invasion and intracellular survival within macrophages are active processes and require the bacteria to be alive. An additional parameter to evaluate would be the difference in immunogenicity between the two strains used in the models. Different strains of MA and indeed other species of mycobacteria stimulate different arms of the immune response to variable magnitudes; therefore we cannot predict that the outcome of either of these exposure models will be replicated if a different NTM isolate is used. Non-pathogenic species of mycobacteria have uncapped lipoarabinomannan (LAM), which can bind to toll-like receptor (TLR) 2 and CD14 to induce phagocyte activation and induction of pro-inflammatory IL-6 and IL-1β [54]. Mannose capping of LAM avoids binding to TLR2 and instead can bind DC-SIGN and induce IL-10 production from dendritic cells (DC) [55]. Different species of MA have variable levels of mannose capping which may in part explain why different NTM isolates activate different arms of the immune response. MA 104 may influence the regulatory phenotype we observed in the MA-oral model simply by having a high level of mannose capped LAM. Killed MA 724-IP enhanced IFNγ production from CD4+ T cells and CD8+ T cells whilst having little effect on the regulatory response which may suggest that MA 724 has a low level of mannose capped LAM. Employing MA 724 in the oral model and MA 104 in the IP model would clarify the importance of strain in the outcomes we observed.

What restricted us from performing the evaluations above was the timeframes required to complete these models. We know that repeated doses of MA after BCG induce a stronger effect on the immune response than a single dose but it may be possible to manipulate the models by reducing the number of doses and time between doses, thus reducing the timeframe required to complete the models to enable faster evaluation of the outstanding questions.

Exposure to NTM is likely to be an important factor in the reduction of BCG efficacy in tropical regions. This study shows that exposure to NTM can generate diverse effects on BCG mediated protection depending on the model of exposure that is used. Humans are likely to be exposed to different NTM species, via different routes of infection and with both live and dead bacteria. There is a need to determine which exposures are detrimental to BCG efficacy, and to define the immunological mechanisms that modify the protective response conferred by BCG. Without understanding the reasons behind the variable efficacy of BCG, we cannot predict whether the efficacy of a novel TB vaccine candidate will be compromised in the same way. A reproducible model of NTM exposure is necessary to evaluate novel TB vaccine candidates during their development. More research is required in this field to ensure we have an efficacious TB vaccine for all populations.

## Ethical approval

Not required.

## Funding

This work was funded by a Wellcome Trust Senior Clinical Research Fellowship awarded to H McShane.

## Competing interest

None.
